# Correlation of Eight (8) Polymorphisms and Their Genotypes with the Risk Factors of Cardiovascular Disease in a Black Elderly Population

**DOI:** 10.3390/cimb46110753

**Published:** 2024-11-08

**Authors:** Joseph Musonda Chalwe, Christa Johanna Grobler, Wilna Hendrika Oldewage-Theron

**Affiliations:** 1 Department of Health Sciences, Vaal University of Technology, Private Bag X021, Vanderbijlpark 1900, South Africa; jheydot@gmail.com; 2 Department of Nutritional Sciences, Texas Tech University (TTU), Lubbock, TX 79409, USA; wilna.oldewage@ttu.edu; 3 Department of Sustainable Food Systems & Development, University of the Free State, Bloemfontein 9300, South Africa

**Keywords:** polymorphisms, molecular level, biomarkers, cardiovascular diseases, South Africa

## Abstract

Single nucleotide polymorphisms (SNPs) have been associated with the development of cardiovascular diseases (CVDs). This study correlated eight SNPs with the risk factors of CVD in a black elderly population. Genotyping was used to detect eight polymorphisms; rs675 (ApoA-IV), rs699 (Angiotensinogen (AGT)), rs247616 and rs1968905 (Cholesteryl ester transfer protein (CETP)), rs1801278 (Insulin receptor substrate 1 (IRS-1)), rs1805087 (Methylenetetrahydrofolate reductase (MTHFR)) and rs28362286 and rs67608943 (Proprotein convertase subtilisin/kexin type 9 (PCSK9)), as well as their genotypes in deoxyribonucleic acid (DNA) extracted from peripheral blood. The cardiovascular risk (CVR) measurements were conducted on a Konelab 20i Thermo Scientific autoanalyzer and an enzyme-linked immunoassay (ELISA) assay. International Business Machines Corporation (IBM)^®^ Statistical Package for the Social Sciences ^®^ (SPSS) version 28 was used for statistical analysis. The heterozygous and homozygous genotypes of the eight polymorphisms were detected with the corresponding CVD risk factors. Subgroup analysis indicated that certain genotype carriers exhibited variations in their concentrations of CVR factors compared to others; however, these differences did not reach statistical significance. For example, carriers of the G genotype of the rs699 polymorphism showed marginally different blood pressure readings compared to the AG genotype carriers. The multiple linear regression analysis indicated that the only significant association was between PCSK9 and the rs28362286 (*p* = 0.029) polymorphism. The findings of our study show that single nucleotide polymorphisms are disseminated across the human genome. The heterozygous and homozygous genotypes of the SNPs require further investigation as they may have independent and possible collective roles in increasing the risk of CVDs.

## 1. Introduction

Cardiovascular diseases (CVDs) are the leading cause of death worldwide. CVDs include several disorders that affect the cardiac muscle and the vascular system supplying blood to the heart, brain and other vital organs [1,2]. Over the years, there has been an epidemiological transition that has led to a constant increase in the prevalence of CVDs in African countries, which has been compounded by underlying conditions like hypertension and diabetes mellitus. At the core of this phenomenon are population-wide changes in demographic, social and economic status [3]. According to the heart and stroke foundation (HSFSA), approximately 215 people died daily from CVDs in 2016 [2]. This presents a huge social and economic burden, especially in developing countries. Unlike most life-threatening conditions, CVDs are preventable and controllable. This can be achieved by early interventions and lifestyle adjustments that may control disease progression [2]. Current evidence has shown that many milestones have been achieved in the study of CVD predictions. However, the effect of the risk factors or biomarkers may be unique to different populations. This is because the onset of CVDs has been linked to complex interactions between genes and environmental factors [4,5,6,7,8,9,10,11,12]. For instance, genetic polymorphisms in the genes of apolipoprotein A-IV (Apo-A4), angiotensin (AGT), cholesteryl ester transfer protein (CETP), insulin receptor substrate 1 (IRS-1), methylenetetrahydrofolate reductase (MTHFR) and PCSK9, amongst others [8], have been identified and reported to have an association with increasing an individual’s susceptibility to developing CVDs. Sliwa et al. [3] mentioned that there are challenges in the planning and implementation of efficient health care in the majority of African countries. This is because of a lack of studies, and minimal resources and investment in genetics research. Genetic polymorphisms have the potential to be used in the prediction and development of treatments for CVDs [7,13]. The aim of this study was to correlate eight polymorphisms and their genotypes with the risk factors of CVD in a black elderly population.

## 2. Materials and Methods

### 2.1. Study Design and Sample

This study utilized a convenience sampling method to gather data from 61 elderly individuals attending a day care center in the peri-urban area of Sharpeville, Vaal region, South Africa, as previously documented [14]. Fasting blood samples were collected within a 2 h window from elderly participants aged 60 years or older who voluntarily attended the Sharpeville day care center and provided informed consent to participate. Individuals unable to provide comprehensive information for the consent process due to conditions such as dementia were excluded from the study.

### 2.2. DNA Extraction and Genotyping

The genomic DNA was extracted from peripheral blood samples using the Quick-DNA™ Miniprep DNA purification kit from Zymo Research in Irvine, CA, USA. The extraction process followed the manufacturer’s instructions as previously reported [14]. A total of 200 μL of each blood sample was added to a 1.5 mL microcentrifuge tube. Then, 200 μL of BioFluid & Cell Buffer (Red) and 20 μL of Proteinase K were added to the same microcentrifuge tube. The contents were mixed using a vortex for 15 s and incubated at 55 °C for 10 min. An amount of 1 volume of Genomic Binding Buffer was added to the digested sample, and the mixture was then mixed for another 15 s. The mixture was transferred to a Zymo-Spin™ IIC-XL Column in a Collection Tube and centrifuged at ≥12,000× *g* for 1 min. The collection tube with the flow through was discarded. 400 μL of the DNA Pre-Wash Buffer was added to the same spin column in a new Collection Tube and centrifuged at ≥12,000× *g* for 1 min. The collection tube was then emptied. The spin column was washed in 2 steps by adding 700 μL and 200 μL g-DNA Wash Buffers to the spin column respectively, with subsequent centrifugation at ≥12,000× *g* for 1 min. The collection tube was then emptied again after each wash. The spin column was transferred to a clean microcentrifuge tube, and 50 μL DNA Elution Buffer was added directly on the matrix. This was followed by incubation for 5 min at room temperature, then centrifugation at maximum speed for 1 min to elute the DNA. After extraction, the DNA concentration was measured using the NanoDrop^®^ 2000 from NanoDrop Technologies in Wilmington, DE, USA, and DNA purity was determined by 1% agarose gel analysis using the A260/A280 ratio. A ratio of ~1.8 is generally accepted as indicating pure DNA.

The genotyping was outsourced to Inqaba Biotechnical Industries (Pty) Ltd., located in Pretoria, South Africa, as previously referenced [14]. They utilized the Agena Bioscience MassARRAY genotyping system, which employs single base extension or cleavage chemistry in conjunction with matrix-assisted laser desorption/ionization-time of flight (MALDI-TOF) mass spectrometry. The SNPs rs675, rs699, rs247616, rs1968905, rs1801278, rs1805087, rs28362286 and rs67608943 were analyzed in each sample. The first step was Polymerase Chain Reaction (PCR), which was carried out using the Bio-Rad CFX Real-Time PCR System, located in Hercules, CA, USA, following the manufacturer’s specifications. The primers and probes were obtained from Inqaba Biotech, located in Pretoria, South Africa, at concentrations of 10 μM. The amplification of the specific genomic DNA fragments was conducted using PCR. A Multiplex PCR cocktail was prepared according to the manufacturer’s instructions. A no-template control was included in every PCR reaction to detect false positive reactions. The products were analyzed using the MassARRAY Compact mass spectrometer and Agena real-time detection software version 1, based in San Diego, CA, USA. This was then followed by genotyping on the Agena MassARRAY platform, based in San Diego, CA, USA. The genotyping was performed using the TaqMan^®^ Pre-designed SNP Genotyping Assay Kit from Thermo Fisher Scientific, located in Foster City, CA, USA. The 20 µL reaction mix consisted of 1 µL of template DNA (15 ng/µL), 10 µL of TaqMan^®^ Genotyping Master Mix (Cat. # 4371355), 1 µL of probe (TaqMan^®^ Pre-designed SNP Genotyping Assay) and 8 µL of deionized water. Before the reaction, the probe was diluted in Tris EDTA buffer (10 mM Tris–HCl (pH 8.0), 0.1 mM EDTA) at a 1:1 ratio. The detection of rs675, rs699, rs247616, rs1968905, rs1801278, rs1805087, rs28362286 and rs67608943 was successful in the participants, and the genotypes of the SNPs were also determined.

### 2.3. Blood Pressure Measurements

Blood pressure (BP) measurements were taken using a previously reported method [15]. Participants were asked to sit quietly for at least 5 min in a chair with back support, feet on the floor, and their arm supported at heart level before the measurements. The Tensoval^®^ duo control monitor (The Hartmaan Group, Heidenheim, Germany) was securely wrapped around the right arm wrist to consistently measure systolic and diastolic BP readings in duplicate on two different days at the same time by a Health Professions Council of South Africa (HPCSA)-registered nurse. To ensure accuracy, the average systolic and diastolic readings were then calculated and recorded. Hypertension was defined according to the South African Hypertension Society (SAHS) guidelines as ≥140/90 mmHg.

### 2.4. Biochemical Measurements

The serum was obtained from the vacutainer blood collection tubes by centrifuging whole blood for 15 min at 4500 rpm, as previously described [14]. The serum aliquots were then stored at −20 °C in the laboratory until analysis. Various components such as serum apolipoprotein A-IV (Apo A-IV), apolipoprotein B (ApoB), high-density lipoprotein cholesterol (HDL-C), homocysteine (hcy), insulin, lipoprotein (a) (Lipo (a)), low-density lipoprotein cholesterol (LDL-C), total cholesterol (TC) and triglyceride (TG) were quantitatively measured using standardized commercial kits from Thermo Fisher Scientific, USA, on a Konelab 20i Thermo Scientific autoanalyzer. This analyzer operates based on colorimetric and immunoturbidimetric principles. Before conducting the tests on the serum samples, lyophilized calibrator and quality control samples from the manufacturer were reconstituted and run to validate the tests. Proprotein convertase subtilisin/kexin type 9 (PCSK9) was assessed using an internationally standardized kit from EIAab^®^ (Wuhan, Hubei Province, China). The kit comprised assay plates with micro-titer wells pre-coated with antibodies specific to PCSK9. Reagent preparation involved reconstituting a wash buffer with distilled water, reconstituting a standard with sample diluent and preparing a working solution of detection reagents A and B with their corresponding assay diluents (Eiaab, Wuhan, Hubei Province, China). The washing steps were carried out using the W206—Microplate Washer (Chengdu Empsun Medical Technology Co., Ltd., Chengdu, Sichuan, China). The final absorbance readings were performed using the Rayto RT-2100C microplate reader (Bio-Asia Diagnostics Co Ltd, Hong Kong, China). The plate was immediately read using the M201—ELISA microplate reader (Chengdu Empsun Medical Technology Co., Ltd., Sichuan, China). Calibration standards were run in duplicate to generate a standard curve for determining serum PCSK9 levels.

### 2.5. Data Analysis

The raw data were cleaned and entered into Microsoft Office Excel and then imported into IBM SPSS version 28 (Statistical Package for the Social Sciences) for statistical analysis, as previously mentioned [14]. All *p*-values were considered two-tailed, and any values less than 0.05 were deemed statistically significant. Means and standard deviations were used for normally distributed data, while medians and interquartile ranges (IQR) were used for data that were not normally distributed. Multilinear regression analysis was conducted to assess the relationship between the polymorphism genotypes and the CVR markers. Eight polymorphisms were examined: rs675 (ApoA-IV), rs699 (Angiotensinogen (AGT)), rs247616 and rs1968905 (CETP), rs1801278 (IRS-1), rs1805087 (MTHFR) and rs28362286 and rs67608943 (PCSK9) due to their influence on the gene activity that affects the concentrations of corresponding CVR markers in blood. We assessed the normal distribution of the residuals, checked for multicollinearity and homoscedasticity and evaluated the priori power to analyze the findings. Subsequently, we utilized a right-tailed F test to assess the statistical significance of the regression model.

## 3. Results

The mean ± SD age of the elderly was 73 ± 9 years. The heterozygous and homozygous genotypes of the eight polymorphisms were successfully detected with the corresponding risk factors of CVD in the elderly population (Table 1). According to the South African Hypertension Society 2019 guidelines, our participants’ mean systolic blood pressure and mean diastolic blood pressure were both above the normal ranges. Additionally, the mean/median serum levels of Apo A-I, ApoB, HDL-C and TG were within the normal reference ranges. The mean PCSK9 levels were lower than the normal reference ranges. Even though a majority of our participants had desirable serum TC (42.6%), HDL-C (27.9%), LDL-C (44.3%) and TG (55.7%), some had high risk levels of the lipid parameters indicating an abnormal lipid profile TC (18%), HDL-C (13.1%), LDL-C (21.3%) and TG (6.6%).

### Association of Genotypes and CVR Factors

The results of the multiple linear regression analysis indicated a significant relationship between the rs28362286 polymorphism and the PCSK9 levels in this elderly population (F(1, 47) = 5.1, *p* = 0.0290, R^2^ = 0.1, R^2^ adjusted = 0.08). This suggests that the rs28362286 polymorphism had a strong direct association with PCSK9 levels. However, the analysis revealed that the other SNPs showed collectively non-significant relationships with cardiovascular risk factors.

## 4. Discussion

In this study, we examined eight polymorphisms: rs675—ApoA-IV, rs699—Angiotensinogen (AGT), rs247616 and rs1968905—CETP, rs1801278—IRS-1, rs1805087—MTHFR and rs28362286 and rs67608943—PCSK9. Subgroup analysis revealed that specific genotype carriers demonstrated differences in the concentrations of cardiovascular risk (CVR) factors in comparison to other groups; nonetheless, these variations did not achieve statistical significance. The outcomes of the multiple linear regression analysis revealed that only the rs28362286 polymorphism exhibited a direct correlation with PCSK9 levels (*p* = 0.029). Conversely, the other single nucleotide polymorphisms (SNPs) demonstrated non-significant associations with cardiovascular risk factors.

PCSK9 is a proteolytic enzyme that plays a crucial role in the regulation of LDL-C indirectly by degrading LDL-C receptors [16]. The main source of PCSK9 is hepatocytes in the liver, but it is also expressed in the intestine and kidneys [17]. This serine protease is encoded by the PCSK9 gene [12]. For this reason, mutations like gain-of-function (GOF) and loss-of-function (LOF) in this gene have been suggested to increase the degradation of LDL-C receptors. This reduces the number of LDL-C receptors which in turn increases the LDL-C concentrations in blood a condition known as hypercholesterolemia [12,18,19,20]. The rs28362286 (C679X) and rs67608943 (Y142X) are loss-of-function (LOF) mutations of the PCSK9 gene. The rs28362286 mutation is also referred to as C679X and the rs67608943 mutation is also known as Y142X [12,21]. In our study, the frequencies of the C and CA genotypes of rs28362286 were 95% and 5% respectively. Interestingly. in the case of rs67608943, all the participants had the C genotype. Even though our population had generally low concentrations of PCSK9, the carriers of the CA genotype of rs28362286 had the highest concentrations compared to the C carriers of both mutations. This is similar to the findings reported by Lakoski et al. [22], where LOF mutations in the PCSK9 gene were linked to low plasma PCSK9 concentrations. We also found that most of our participants had desirable serum TC, HDL-C, LDL-C and TG levels, while some had high risk levels of the lipid parameters suggesting hypercholesterolemia. Studies of these mutations have been associated with hypocholesterolemia in various populations [23,24,25,26]. This might explain why the majority of our participants had desirable TC, HDL-C, LDL-C and TG levels. Interestingly, the rs28362286 LOF mutation has also been reported to reduce fasting glucose levels in adolescents and adult populations [27,28]. These researchers have indicated that these LOF mutations warrant further investigations as they may have varied effects on metabolic biomarkers. Therefore, people with dyslipidemia and other underlying conditions such as diabetes mellitus or CVR should avoid consuming a high cholesterol diet [29]. Additionally, further studies on the regulation of these mutations may lead to the development of efficient PCSK9 inhibitor-based treatments for these conditions [23].

Current evidence, especially in the field of nutrigenomics, has indicated the need to identify genetic risk factors of conditions like cardiovascular disease in order to enable the production and improvement of preventive and therapeutic solutions. This is because CVDs can be attributed to complex interactions between genes (Apo-A4, AGT, CEPT, IRS1, MTHFR and PCSK9, amongst others) and environmental factors (tobacco smoke, unhealthy diet, lack of physical activity). These genes have been identified as critical players in the onset of these diseases [4,5,6,8,9,10,11,12].

A strength of our study is that to our knowledge, this report is the first of its kind to be conducted on eight genetic polymorphisms and their corresponding CVD risk factors in an African population. It supplements information obtained from studies that also investigated polymorphisms that are associated with an increased risk of CVDs. This is valuable information for genetic risk profiling that can be used by policy makers to develop targeted treatment and early prevention models for CVDs [30].

Our research was limited to participants who visited the elderly day care center in Sharpeville. We specifically focused on examining eight polymorphisms: rs675 (ApoA-IV), rs699 (Angiotensinogen (AGT)), rs247616 and rs1968905 (CETP), rs1801278 (IRS-1), rs1805087—(MTHFR) and rs28362286 and rs67608943 (PCSK9). Similarly, in assessing the cardiovascular risk (CVR) factors, we only analyzed Apo A-IV, ApoB, HDL-C, hcy, insulin, Lipo (a), LDL-C, TC and TG. Lastly, the use of a convenient sample method resulted in a small population size, impacting the generalizability of our findings. Consequently, the findings of this report are specific to this particular setting and cannot be applied to the broader population.

## 5. Conclusions

The current study could enable the identification of distinctive combinations or sets of polymorphisms that are linked to CVDs precisely in the black elderly population from Sharpeville. The results of our study show that the heterozygous and homozygous genotypes of the eight polymorphisms may have independent and possible collective roles in increasing the risk of CVDs in this elderly population. Larger studies with different populations are recommended to confirm the results.

## Figures and Tables

**Table 1:** Association between polymorphism genotypes and risk factors for cardiovascular disease.

SNP Genotypes	Risk Factors	Association (*p*-Value)
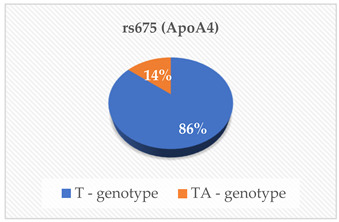	ApoA1 (g/L) ApoB (g/L) Lipo (a) (nmol/L)	0.971 0.857 0.536
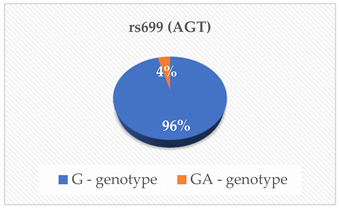	BP (mmHg)	0.607
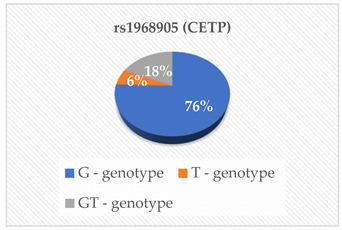	ApoA1 (g/L) ApoB (g/L) TC (mmol/L) TG (mmol/L) LDL-C (mmol/L) HDL-C (mmol/L) Lipo (a) (nmol/L)	0.780 0.364 0.291 0.380 0.654 0.694 0.237
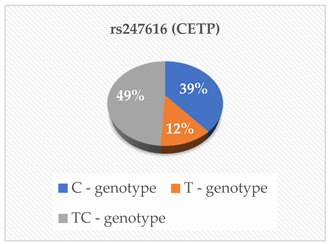	ApoA1 (g/L) ApoB (g/L) TC (mmol/L) TG (mmol/L) LDL-C (mmol/L) HDL-C (mmol/L) Lipo (a) (nmol/L)	0.308 0.860 0.713 0.505 0.707 0.482 0.466
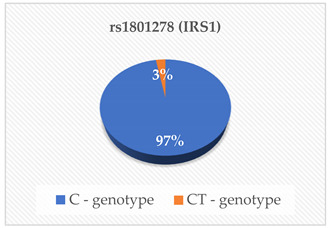	Glucose (mmol/L) Insulin (μU/Ml)	0.201 0.322
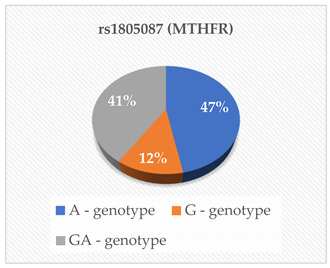	Hcy (μmol/L)	0.835
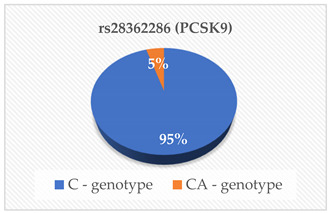	PCSK9 (ng/mL)	0.029
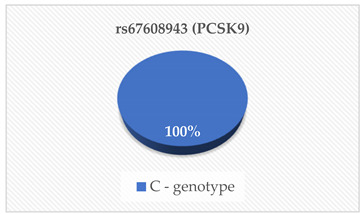	PCSK9 (ng/mL)	<0.001

Abbreviations: AGT, Angiotensinogen; ApoA-IV, Apolipoprotein A-IV; ApoB, apolipoprotein B; CETP, Cholesteryl ester transfer protein; HDL-C, high-density lipoprotein cholesterol; IRS-1, Insulin receptor substrate-1; Lipo (a), Lipoprotein (a); LDL-C, low-density lipoprotein cholesterol; MTHFR, Methylenetetrahydrofolate reductase; PCSK9, Pro-protein convertase subtilisin/kexin type 9, *p*, *p*-value, SD, standard deviation; SNP, single-nucleotide polymorphism; TC, total cholesterol; TG, triglyceride. The T, TA, G, AG, G, T, GT, C, T, TC, C, CT, A, G, GA, C, CA and C respectively, indicate the SNP genotypes.

## Data Availability

The original contributions presented in the study are included in the article, further inquiries can be directed to the corresponding author.

## References

[1] Gaziano T., Reddy K.S., Paccaud F., Horton S., Chaturvedi V., Jamison D.T., Breman J.G., Measham A.R., Alleyne G., Claeson M., Jamison D.T., Breman J.G., Measham A.R., Alleyne G., Claeson M., Evans D.B., Jha P., Mills A., Musgrove P. (2006). Cardiovascular Disease. Disease Control Priorities in Developing Countries.

[2] Byrne J., Eksteen G., Crickmore C. (2016). South Africa Cardiovascular Disease Statistics Reference Document.

[3] Sliwa K., Acquah L., Gersh B.J., Mocumbi A.O. (2016). Impact of Socioeconomic Status, Ethnicity, and Urbanization on Risk Factor Profiles of Cardiovascular Disease in Africa. Circulation.

[4] Heianza Y., Qi L. (2019). Impact of Genes and Environment on Obesity and Cardiovascular Disease. Endocrinology.

[5] Moreau J.L.M., Kesteven S., Martin E.M.M.A., Lau K.S., Yam M.X., O’reilly V.C., Del Monte-Nieto G., Baldini A., Feneley M.P., Moon A.M. (2019). Gene-environment interaction impacts on heart development and embryo survival. Development.

[6] Brennan L., De Roos B. (2021). Nutrigenomics: Lessons learned and future perspectives. Am. J. Clin. Nutr..

[7] Chalwe J.M., Grobler C., Oldewage-Theron W., Chahine J. (2021). Genetic Polymorphisms and Their Interactions with the Risk Factors of Cardiovascular Diseases: Review Chapter. Risk Factors for Cardiovascular Disease.

[8] National Center for Biotechnology Information APOB Apolipoprotein B [Homo Sapiens (Human)]. https://www.ncbi.nlm.nih.gov/gene?Db=gene&Cmd=DetailsSearch&Term=338.

[9] National Center for Biotechnology Information CETP Cholesteryl Ester Transfer Protein [Homo Sapiens (Human)]. https://www.ncbi.nlm.nih.gov/gene?Db=gene&Cmd=DetailsSearch&Term=1071.

[10] National Center for Biotechnology Information LDLR Low Density Lipoprotein Receptor [Homo Sapiens (Human)]. https://www.ncbi.nlm.nih.gov/gene?Db=gene&Cmd=DetailsSearch&Term=3949.

[11] National Center for Biotechnology Information MTHFR Methylenetetrahydrofolate Reductase [Homo Sapiens (Human)]. https://www.ncbi.nlm.nih.gov/gene?Db=gene&Cmd=DetailsSearch&Term=4524.

[12] National Center for Biotechnology Information PCSK9 Proprotein Convertase Subtilisin/Kexin Type 9 [Homo Sapiens (Human)]. https://www.ncbi.nlm.nih.gov/gene/255738.

[13] Francula-Zaninovic S., Nola I.A. (2018). Management of Measurable Variable Cardiovascular Disease’ Risk Factors. Curr. Cardiol. Rev..

[14] Chalwe J.M., Grobler C., Oldewage-Theron W. (2023). Development of a Structural Equation Model to Examine the Relationships between Genetic Polymorphisms and Cardiovascular Risk Factors. Nutrients.

[15] Chalwe J.M., Mukherjee U., Grobler C., Mbambara S.H., Oldewage-Theron W. (2021). Association between hypertension, obesity and dietary intake in post-menopausal women from rural Zambian communities. Health SA Gesondheid.

[16] Singh K., Gupta J.K., Kumar S., Singh K., Meenakshi K., Kumar K. (2022). PCSK9 Inhibitors: Pharmacology and Therapeutic Potential. Preprints.

[17] Shapiro M.D., Tavori H., Fazio S. (2018). PCSK9: From Basic Science Discoveries to Clinical Trials. Circ. Res..

[18] Cariou B., Maya C.L., Costet P. (2011). Clinical aspects of PCSK9. Atherosclerosis.

[19] Nussbaum R.L., Mcinnes R.R., Willard H.F., Nussbaum R.L. (2016). The Molecular, Biochemical, and Cellular Basis of Genetic Disease. Thompson & Thompson Genetics in Medicine.

[20] Seidah N.G., Prat A. (2022). The Multifaceted Biology of PCSK9. Endocr. Rev..

[21] Tsai C.-W., North K.E., Tin A., Haack K., Franceschini N., Saroja Voruganti V., Laston S., Zhang Y., Best L.G., Maccluer J.W. (2015). Both rare and common variants in PCSK9 influence plasma low-density lipoprotein cholesterol level in American Indians. J. Clin. Endocrinol. Metab..

[22] Lakoski S.G., Lagace T.A., Cohen J.C., Horton J.D., Hobbs H.H. (2009). Genetic and Metabolic Determinants of Plasma PCSK9 Levels. J. Clin. Endocrinol. Metab..

[23] Hooper A.J., Marais A.D., Tanyanyiwa D.M., Burnett J.R. (2007). The C679X mutation in PCSK9 is present and lowers blood cholesterol in a Southern African population. Atherosclerosis.

[24] Huang C.C., Fornage M., Lloyd-Jones D.M., Wei G.S., Boerwinkle E., Liu K. (2009). Longitudinal association of PCSK9 sequence variations with low-density lipoprotein cholesterol levels: The Coronary Artery Risk Development in Young Adults Study. Circulation. Cardiovasc. Genet..

[25] Kent S.T., Rosenson R.S., Avery C.L., Chen Y.-D.I., Correa A., Cummings S.R., Cupples L.A., Cushman M., Evans D.S., Gudnason V. (2017). PCSK9 Loss-of-Function Variants, Low-Density Lipoprotein Cholesterol, and Risk of Coronary Heart Disease and Stroke. Circulation. Cardiovasc. Genet..

[26] Krittanawong C., Khawaja M., Rosenson R.S., Amos C.I., Nambi V., Lavie C.J., Virani S.S. (2022). Association of PCSK9 Variants With the Risk of Atherosclerotic Cardiovascular Disease and Variable Responses to PCSK9 Inhibitor Therapy. Curr. Probl. Cardiol..

[27] Chikowore T., Cockeran M., Conradie K.R., van Zyl T. (2018). C679X loss-of-function PCSK9 variant lowers fasting glucose levels in a black South African population: A longitudinal study. Diabetes Res. Clin. Pract..

[28] Chikowore T., Sahibdeen V., Hendry L.M., Norris S.A., Goedecke J.H., Micklesfield L.K., Lombard Z. (2019). C679X loss-of-function PCSK9 variant is associated with lower fasting glucose in black South African adolescents: Birth to Twenty Plus Cohort. J. Clin. Transl. Endocrinol..

[29] Chopra A.K. (2024). Dietary management of dyslipidemia. Indian Heart J..

[30] Giglio R.V., Stoian A.P., Patti A.M., Rizvi A.A., Sukhorukov V., Ciaccio M., Orekhov A., Rizzo M. (2021). Genetic and Epigenetic Biomarkers for Diagnosis, Prognosis and Treatment of Metabolic Syndrome. Curr. Pharm. Des..

